# Supplementary material for: Evolution End Classification of tfd Gene Clusters Mediating Bacterial Degradation of 2,4-Dichlorophenoxyacetic Acid (2,4-D)
Source: Int J Mol Sci. 2023 Sep 21;24(18):14370. doi: 10.3390/ijms241814370 (PMC10531765; doi:10.3390/ijms241814370)
Supplement: Supplementary file 1 [file ijms-24-14370-s001.zip › Table S1.pdf]

Table 1. List of *tfd*, *tcb* and *clc* gene clusters

Structure of clusters (proposed nomenclature)	Structure of clusters (authors version)	Strain (plasmid)	GenBank accession no.	Reference
$tfd_{I}BFEDCT$, $tfd_{II}KBFECDR$, $tfd_{II}SA$	$tfdB_{I}F_{I}E_{I}D_{I}C_{I}T$, $tfdKB_{II}F_{II}E_{II}C_{II}D_{II}R$, *tfdSA*	*Cupriavidus pinatubonensis* JMP134 (pJP4)	AY365053	[18]
$tfd_{I}BFEDCT$, $tfd_{I}BFED$, $tfd_{II}SA$, $tfd_{II}AKBFECDR$	*tfdBFET*, *tfdSA*, *tfdAKBFECDR*, *tfdBFEDC*	*Cupriavidus oxalaticus* X32 (unnamed5 - plasmid5 (plas5))	(CP038640)	[5]
$tfd_{I}BFEDCT$, $tfd_{II}KFECD$, $tfdA_{II}$, $tfdS_{II}$	$tfdB_{I}F_{I}E_{I}D_{I}C_{I}T$, $tfdKFE_{II}C_{II}D_{II}$, *tfdA*, *tfdS*	*Cupriavidus* sp. STW8-1 (pPO1)	CCJH010000003, CCJH010000006, CCJH010000007	[44]
$tfd_{I}BFEDCT$, $tfd_{II}FECDR$, $tfdA_{II}$, $tfdS_{II}$	$tfdB_{I}F_{I}E_{I}D_{I}C_{I}T$, $tfdKFE_{II}C_{II}D_{II}R$, *tfdA*, *tfdS*	*Cupriavidus* sp. TGCL-2 (pPO2)	CCJI010000002, CCJI010000001, CCJI010000004	
$tfd_{I}BFEDCT$, $tfd_{II}FECD$, $tfd_{II}SA$	$tfdB_{I}F_{I}E_{I}D_{I}C_{I}T$, $tfdKFE_{II}C_{II}D_{II}$, *tfdSA*	*Cupriavidus* sp. TGCL-3 (pPO3)	CCJJ010000002, CCJJ010000003, CCJJ010000004	
$tfd_{I}BFEDCT$, $tfd_{II}SA$	$tfdB_{I}F_{I}E_{I}D_{I}C_{I}T$, *tfdSA*	*Burkholderia* sp. STW8-4 (pPO4)	CCJK010000001, CCJK010000002	
$tfd_{I}BFEDCT$, $tfd_{II}FECDR$, $tfdA_{II}$, $tfdS_{II}$	$tfdB_{I}F_{I}E_{I}D_{I}C_{I}T$, $tfdKFE_{II}C_{II}D_{II}R$, *tfdA*, *tfdS*	*Cupriavidus* sp. STW8-7 (pPO7)	CCJL010000002, CCJL010000003, CCJL010000006, CCJL010000004	
$tfd_{I}BFEDCT$, $tfd_{II}FECDR$, $tfd_{II}SA$	$tfdB_{I}F_{I}E_{I}D_{I}C_{I}T$, $tfdKFE_{II}C_{II}D_{II}R$, *tfdSA*	*Cupriavidus* sp. STW8-10 (pPO10)	CCJM010000004, CCJM010000001	

$tfd_{I}BFEDCT$, $tfd_{II}FECDR$, $tfd_{II}A$, $tfd_{II}S$	$tfdB_{I}F_{I}E_{I}D_{I}C_{I}T$, $tfdKFE_{II}C_{II}D_{II}R$, *tfdA*, *tfdS*	*Ralstonia* sp. TGCL-16 (pPO16)	CCJN010000003, CCJN010000004, CCJN010000006	
$tfd_{I}BFEDCT$, $tfd_{II}KBFECDR$, $tfdA_{II}$, $tfdS_{II}$	$tfdB_{I}F_{I}E_{I}D_{I}C_{I}T$, $tfdKB_{II}FE_{II}C_{II}D_{II}R$, *tfdA*, *tfdS*	*Cupriavidus* sp. TGCL-26 (pPO26)	CCJO010000003, CCJO010000004, CCJO010000005	
$tfd_{I}BFEDCT$, $tfd_{II}FECDR$, $tfdA_{II}$, $tfdK_{II}$, $tfdS_{II}$	$tfdB_{I}F_{I}E_{I}D_{I}C_{I}T$, $tfdKFE_{II}C_{II}D_{II}R$, *tfdA*, *tfdS*	*Burkholderia* sp. TGCL-27 (pPO27)	CCEI010000001, CCEI010000004, CCEI010000005	
$tfd_{II}AKBFECDR$	-*	Soil sample, uncultured bacterium (pEMT3)	JX469827	[55]
$tfd_{II}AKBFECDR$	*tfdRDCEFBKA*	*Ralstonia pickettii* 712 (p712)	JQ436722	[40]
$tfd_{II}AKBFECDR$	*tfdRDCEFBKA*	*Variovorax* sp. DB1 (pDB1)	JQ436721	
$tfd_{I}BFEDCT$, $tfd_{II}SA$	-	*Paraburkholderia hospita* DSM 17164 (pEMT1)	CP026110	[57]
$tfd_{I}FEDCT$	*tfdT-CDEF*	*Burkholderia* sp. NK8	AB050198	[64]
$tfd_{I}FEDCT$	*tfdCDEF*	*Caballeronia* sp. NK8 (previously *Burkholderia* sp. NK8) (pNK84)	AP024328	[45]
$tfd_{I}FEDCT$	*tfdFEDCT*	*Paraburkholderia phytofirmans* OLGA172 (chromosome 1) (=*Burkholderia* sp. OLGA172 = *Burkholderia* sp. R172)	CP014578	[43]
$tfd_{I}DCTT$	*tfdT1T2CD*		AY168634	unpublished
$tfd_{I}FEDC$	*tfdCDEF*	Soil sample, uncultured bacterium	AB478351	[58]
$tfd_{I}BFEDCT$, $tfd_{II}SA$, $tfd_{III}FAKBECDR$, $tfd_{III}BECRR$	-	*Burkholderia* sp. KK1 (pkk4)	CP016005	unpublished

$tfd_{\mathrm{III}}FAKBECDR$, $tfd_{\mathrm{III}}BECR$	$tfdFAKB_{\mathrm{I}}E_{\mathrm{I}}C_{\mathrm{I}}D_{\mathrm{I}}R_{\mathrm{I}}$, $tfdC_{\mathrm{II}}E_{\mathrm{II}}B_{\mathrm{II}}$	*Burkholderia* sp. M701 (pM7012)	AB853026	[42]
$tfd_{\mathrm{III}}F,AKBECR$, *tcbD*ORF3	*tfdDRCEBKAF*, *tfdDI*	*Burkholderia cepacia* 2a (pIJB1)	AF029344, JX847411	[36]
$tfd_{\mathrm{III}}F,AKBECR$, *tcbD*ORF3, *tcbD*ORF3	*tfdD*, *orf5*, *tfdRCEBKA*, *tfdF*	*Achromobacter xylosoxidans subsp. denitrificans* EST4002 (pEST4011)	AY540995	[37]
$tfd_{\mathrm{III}}AKBECR$, *tcbD*ORF3	*tfdAKBECR*, *tfdD*	Agricultural soil, uncultured bacterium (pAKD25)	JN106170	[39]
$tfd_{\mathrm{III}}AKBECR$	*tfdAKBECR*	Agricultural soil, uncultured bacterium (pAKD26)	JN106171	
$tfd_{\mathrm{III}}KBECR$, *tcbD*ORF3	*tfdA*, *tfdKBECR*, *tfdD*	*Variovorax paradoxus* TV1 (pTV1)	VPU65531, AB028643	[61]
tcbFEDCR , $tfd_{\mathrm{III}}AKBEtcbCR$	*tfdRCDEF*, $tfdRC_{\mathrm{II}}E_{\mathrm{II}}BKA$	*Delftia acidovorans* P4a	AY078159	[59]
tcbFEDCR , $tfd_{\mathrm{III}}BECR$	-	*Cupriavidus basilensis* DSM 11853 (pRK1-5)	CP062809	[60]
$tfd_{\mathrm{IV}}CEDRF,B$	*tfdBaFRDEC*	*Bradyrhizobium* sp. RD5-C2	BOVL01000048	[10]
$tfd_{\mathrm{IV}}CE,D,R,F$	*tfdF,S,D,EC*	*Sphingomonas histidinilytica* BT1 5.2	WMBU01000019	[7]
$tfd_{\mathrm{IV}}RD,B,CE$	*tfdD,B,CE*	*Sphingomonas histidinilytica* BT1 5.2	WMBU01000047	
$tfd_{\mathrm{I}}BFEDCT$	*tfdBFEDCS*	*Bordetella petrii* BT1 9.2	WMBV01000066	
$tfd_{\mathrm{IV}}ECFRD,B$	*tfdBCDEFKR*	*Sphingomonas* sp. ERG5 (pCADAB1)	KF494257	[41]
$tfd_{\mathrm{IV}}ECFRD,B$	$dccEA_{\mathrm{I}}D_{\mathrm{I}}$, *tfdB*, *tfdK*, $dccA_{\mathrm{II}}D_{\mathrm{II}}$ $dccEA_{\mathrm{I}}D_{\mathrm{I}}$	*Sphingobium herbicidovorans* MH	AJ628862, AJ628863	[62]

	tfdDRFCE, *tfdB*		CP020538	[63]
tfd$_{\mathrm{IV}}$*FRD,B,CE*	*tfdDRF*, *tfdB*, *tfdC2E2*	*Sphingobium herbicidovorans* MH (pMSHV)	CP020539	
tfd$_{\mathrm{IV}}$*ECFRD,B*	*tfdEICIFIRDI,K,BI*	*Sphingopyxis* sp. DBS4 (pDB-1)	CP102385	[46]
tfd$_{\mathrm{IV}}$*D,FEC,B*	*tfdDII,FIIEIICII,BII*	*Sphingopyxis* sp. DBS4 (pDB-4)	CP102388	
tfd$_{\mathrm{IV}}$*ECFRD,B*	-	*Sphingopyxis* sp. KK2	(LYVN01000006)	unpublished
tfd$_{\mathrm{IV}}$*ECFRD,B*	*tfdDRFCE*, *tfdB*	*Sphingomonas* sp. TFD44	-	[63]
tfd$_{\mathrm{IV}}$*ECFRD*	*tfdDRFCE*		AY598949	[38]
tfd$_{\mathrm{IV}}$*FEC*	*tfdC2E2F2*		AY598950	
tfd$_{\mathrm{IV}}$*D,FEC*	-	*Tardibacter chloracetimidivorans* JJ-A5 (*Sphingomonas* sp. JJ-A5) (pHSL1)	CP018222	unpublished
tfd$_{\mathrm{IV}}$*D,FEC*	*cnbCDEF*	*Pseudomonas stutzeri* ZWLR2-1	GU181397	[64]
tcbFEDCR	*tcbCDEF*	*Pseudomonas* sp. P51 (pP51)	M57629	[30]
tcbFEDCR	*mocpRABCD*	*Achromobacter xylosoxidans* A8 (pA81)	AJ515144, CP002288	[73]
tcbFEDCR	-	*Sphingomonas* sp. C8-2	CP034357	[56]
tcbFEDCR	*cbnR-ABCD*	*Cupriavidus necator* NH9 (pENH91)	AB019032, CP017760	[48-49]

tcbFEDCR	-	*Cupriavidus basilensis* DSM 11853 (pRK1-5)	CP062809	[60]
tcbFEDCR	*tetRtetCDEF*	*Pseudomonas chlororaphis* RW71	AJ271325	[47]
tcbFEDCR	-	*Delftia acidovorans* CA28 (pC1-1)	HQ891317	unpublished
tcbFEDCR	-	*Bordetella petrii* DSM 12804	AM902716	[54]
tcbFEDCR	*tfdRCDEF*	*Delftia acidovorans* P4a	(AY078159)	[59]
tcbFEDCR	*mocpDCBAR*	Uncultured bacterium (pAKD26)	(JN106171)	[39]
clcEDBAR	*clcABD*	*Escherichia coli* JM103 (pAC27)	M16964	[25]
clcEDBAR	*clcr1a1b1d1e1*	*Pseudomonas knackmussii* B13 (previously *Pseudomonas* sp. strain B13)	HG322950	[52]
	clcRABDE		AJ617740	[65-66]
clcEDBAR	-	*Bordetella petrii* DSM 12804	AM902716	[54]
clcEDBAR	*clcRABDE*	*Paraburkholderia xenovorans* LB400 chromosome 1	CP000270	[67]
	-		CP008760	[68]
clcEDBAR	-	*Pseudomonas aeruginosa* strain JB2 chromosome	CP028917	[71]
	clcRABDE		AF087482	[69]

	clcRABDE		AF164958	[70]
clcEDBAR	*clcRABDE*	*Pseudomonas aeruginosa* 142	AF161263	
clcEDBAR	*clcRABCDE*	*Pandoraea pnomenusa* MCB032 (previously *Pandoraea* sp. strain MCB032)	EF600715	[51]
	-		CP015373	[72]
clcEDBAR	*clcRABCD*	*Alcaligenes* sp. NyZ215	EF544605	[50]
clcEDBAR	*dccEDCBAR*	*Diaphorobacter* sp. strain JS3051	CP065406	[53]
clcEDBAR	-	*Pandoraea* sp. XJJ-1	CP113828	Unpublished
clcEDBAR	*tfdFE(tctC)DCS*	*Achromobacter xylosoxidans* strain BT1 10.2	WMBW01000025	[7]

* sequenced, but not annotated and described
