# Supplementary figures and images for: Evolution End Classification of tfd Gene Clusters Mediating Bacterial Degradation of 2,4-Dichlorophenoxyacetic Acid (2,4-D)
Source: Int J Mol Sci. 2023 Sep 21;24(18):14370. doi: 10.3390/ijms241814370 (PMC10531765; doi:10.3390/ijms241814370)

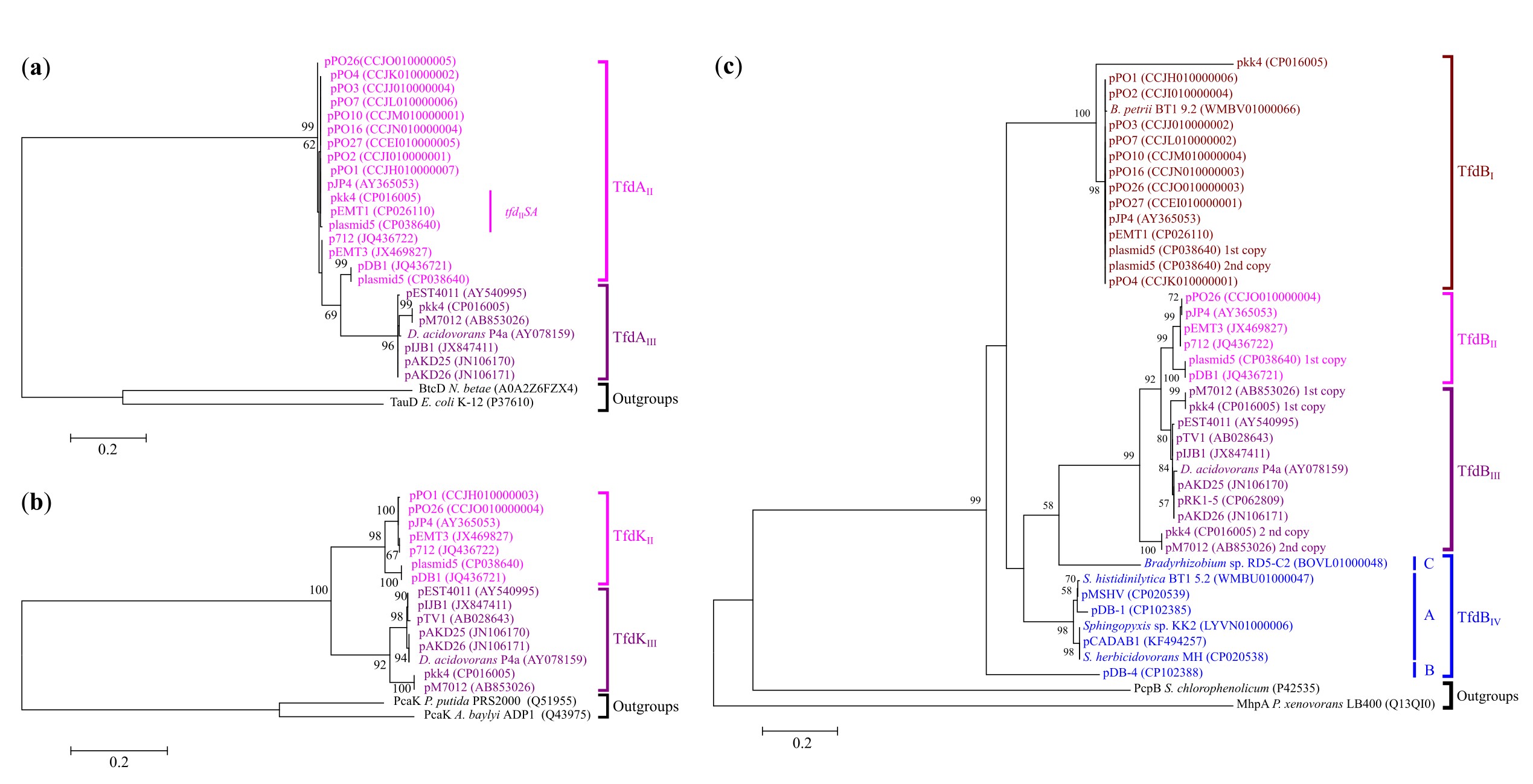

Supplement: Supplementary file 1 [file ijms-24-14370-s001.zip › Figure S1.jpg]

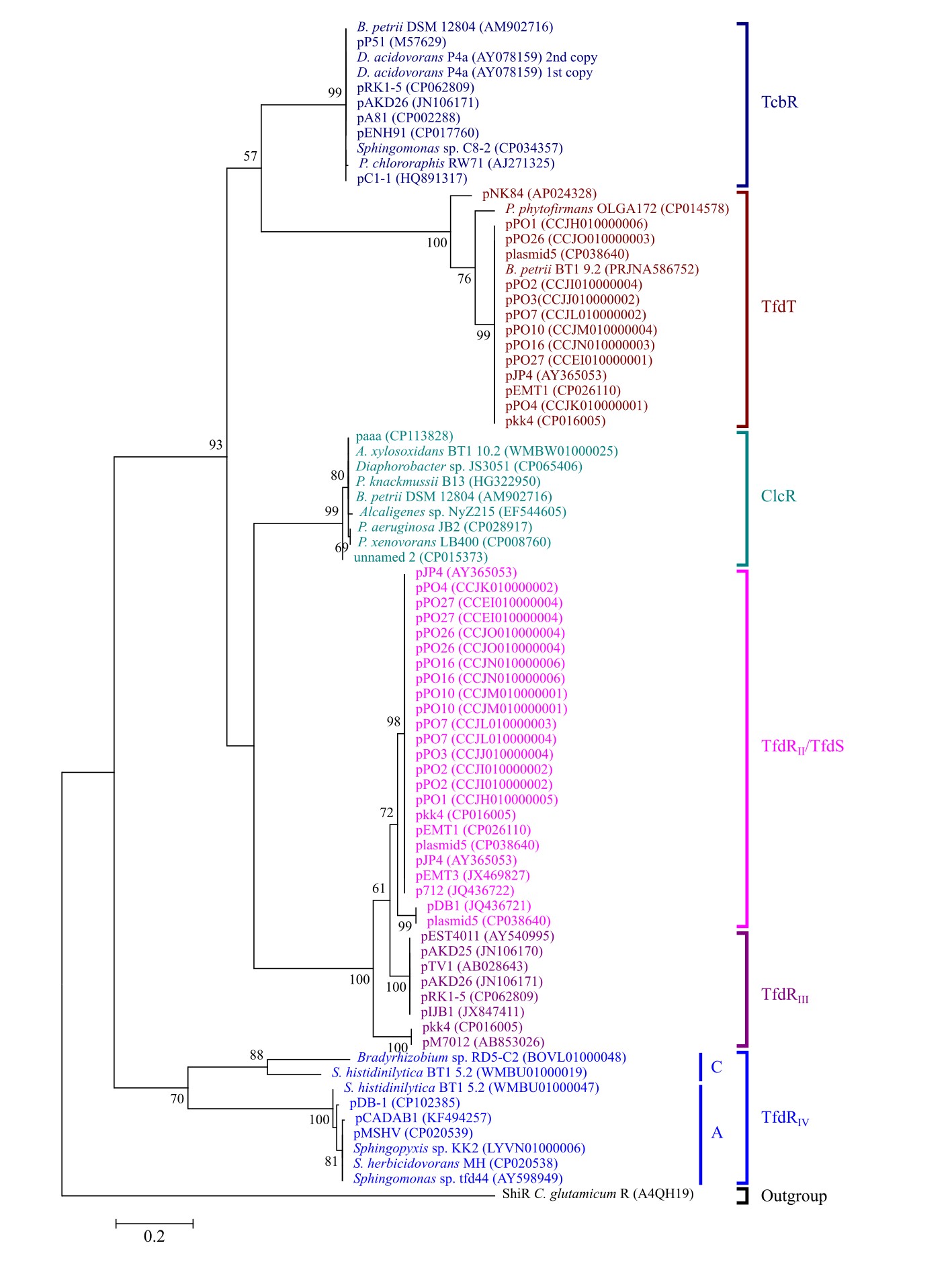

Supplement: Supplementary file 1 [file ijms-24-14370-s001.zip › Figure S2.jpg]
